# A Review of Techniques for Detection of Movement Intention Using Movement-Related Cortical Potentials

**DOI:** 10.1155/2015/346217

**Published:** 2015-12-31

**Authors:** Aqsa Shakeel, Muhammad Samran Navid, Muhammad Nabeel Anwar, Suleman Mazhar, Mads Jochumsen, Imran Khan Niazi

**Affiliations:** ^1^Human Systems Lab, Department of Biomedical Engineering and Sciences, School of Mechanical and Manufacturing Engineering, National University of Sciences and Technology (NUST), Islamabad 44000, Pakistan; ^2^BiSMiL Lab, Computer Science Department, Information Technology University, Lahore 54000, Pakistan; ^3^Center for Sensory-Motor Interaction, Department of Health Science and Technology, Aalborg University, 9100 Aalborg, Denmark; ^4^Center for Chiropractic Research, New Zealand College of Chiropractic, 1060 Auckland, New Zealand; ^5^Faculty of Health & Environmental Sciences, Health & Rehabilitation Research Institute, Auckland University of Technology, 1010 Auckland, New Zealand

## Abstract

The movement-related cortical potential (MRCP) is a low-frequency negative shift in the electroencephalography (EEG) recording that takes place about 2 seconds prior to voluntary movement production. MRCP replicates the cortical processes employed in planning and preparation of movement. In this study, we recapitulate the features such as signal's acquisition, processing, and enhancement and different electrode montages used for EEG data recoding from different studies that used MRCPs to predict the upcoming real or imaginary movement. An authentic identification of human movement intention, accompanying the knowledge of the limb engaged in the performance and its direction of movement, has a potential implication in the control of external devices. This information could be helpful in development of a proficient patient-driven rehabilitation tool based on brain-computer interfaces (BCIs). Such a BCI paradigm with shorter response time appears more natural to the amputees and can also induce plasticity in brain. Along with different training schedules, this can lead to restoration of motor control in stroke patients.

## 1. Introduction

The idea of predicting the motor tasks was initially presented by Helmholtz in 1867. Later on, in the fifties Sperry and Von Holst expressed that motor commands make an internal replica which uncovers the anticipated movement and its subsequent sensations [1–3]. From that point forward, the thought of predicting the results of motor tasks by humans has risen as a conspicuous theory in all features of sensorimotor commands.

The brain's current motor activity can be understood in real time through EEG, which can be further employed for prediction of the next voluntary motor task. Real-time EEG might present novel nonmuscular control channel Brain Computer Interfaces (BCIs) for delivering messages and commands to the external world [4]. The immediate objective of BCIs is to provide completely paralyzed users with basic communication capabilities and determine their intent from a range of different electrophysiological signals [4]. Furthermore, research has demonstrated great prospective in the study of brain rhythms and event-related potentials (ERPs) recorded by EEG. Therefore, understanding and analysis of the brain rhythms and ERPs can be used to predict the future motor activity and can be utilized for rehabilitation of physically impaired persons [5].

Studies have shown that EEG comprises enough real-time information to be utilized for different purposes/tasks such as internet browsing, controlling environment (e.g., light, television, and temperature), word processing, controlling a two-dimensional cursor movement on screen, or even operating neuroprosthesis [4]. Tasks can be designed which can be used for neurorehabilitation of patients affected with neurodegenerative diseases such as amyotrophic lateral sclerosis [6] and other traumatic brain disorders like stroke [7].

The concept of “premovement” or “before the movement” indicates the time when no muscle movement is evident or is unrelated if it occurs, but the subject is fully familiar with the action he is going to perform in the near future. This is also referred to as planning/preparation of the movements. In this time interval (i.e., 0.5–2 s prior to the movement onset), the cortex is adapted for implementation of action [8, 9].

This paper aims to review the different studies which have used movement-related cortical potentials (MRCPs) to predict the upcoming movements. In the next section, we illustrate the key modifications in the EEG data reported prior to the voluntary movement and how the knowledge of these variations can be used to extract information about the forthcoming movement. In each case, we discuss the main foundations of the study and evaluate the EEG setup and protocols. Finally, in the Conclusion, we recapitulate the key ideas with the hope to bring more consideration to the affluence of premovement and premotor imagery EEG.

## 2. Detectable Changes in Brain Rhythm before Onset of Movement

In this section, we summarize the reported changes in EEG prior to the onset of the actual or imagery movement. All the following phenomena have been delineated both when the movement is imagined and when it is actually executed. One or an amalgamation of these progressions is the fundamental spotlight of the studies acquiring features from premotor imagery or premovement period, discussed in Section 3.

### 2.1. MRCP and Its Components

**Figure 1:**
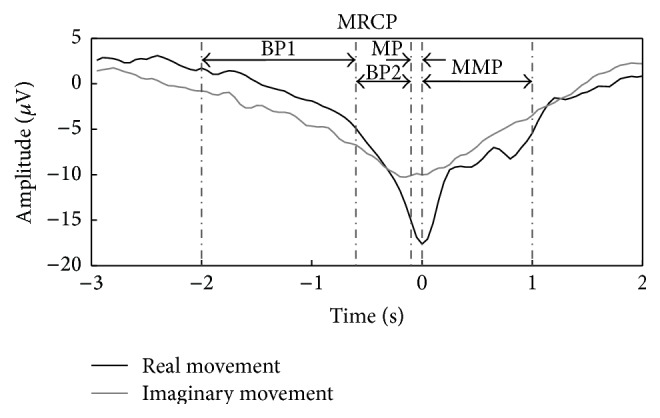
MRCPs of a healthy subject for real and imaginary right ankle dorsiflexion. Each wave is an average of 50 large Laplacian spatial filtered EEG trials recorded from sites F3, Fz, F4, C3, Cz, C4, P3, Pz, and P4. Time 0 s is defined as the movement onset. BP1 is early BP, BP2 is late BP, MP is motor potential, and MMP is movement-monitoring potential. For more information on experiment protocol, see [23].

The implementation of a motor task in humans measured over the primary motor cortex is preceded by a slow decrease in the EEG amplitude (within at least 500 ms) and this potential is known as an MRCP [10], as shown in Figure 1. The MRCP produced in corporation with the planning and execution of a cue-based movement is known as contingent negative variation (CNV) [11], and the one generated in response to self-paced movement is known as Bereitschaftspotential (BP) [12, 13]. The MRCP is present in real as well as in imaginary volitional movements [10]. The MRCP comprises three events called readiness potential (RP) or BP, motor potential, and movement-monitoring potential (MMP), which are thought to reflect movement planning/preparation, execution, and control of performance, correspondingly [14, 15]. The MRCP has been further investigated in normal persons as well as in patients diagnosed with Amyotrophic Lateral Sclerosis, tremor, Parkinson's disease, and stroke, supporting the execution of their motor tasks [14, 16–18]. MRCPs associated with imaginary tasks make them useful for rehabilitation in patients obstructed in movements but still with the ability to wish and imagine a movement [19, 20].

#### 2.1.1. Bereitschaftspotential

BP or RP is a negative cortical potential which starts to grow around 1.5 to 1 s prior to the onset of a voluntary movement [12, 13]. It has two fundamental segments: the first part is a slow-rising negative segment which develops about 1.5 s before the movement onset, known as “early BP,” and is more distinguished in the central-medial scalp, while the second part has a steeper slope and happens around 400–500 ms before the movement onset and is called “late BP” which has maximum amplitude over the primary motor cortex [10]. The start of BP regarding the movement onset varies considerably among different conditions of movement and among subjects [10]. More details on BP can be found in the comprehensive book “The Bereitschaftspotential-Movement Related Cortical Potentials” [21].

#### 2.1.2. Contingent Negative Variation

CNV is a slow negative wave that originates in the interval (1–1.5 s) between a “Warning” and a “Go” stimulus [11]. It shows expectancy for an imminent signal and preparation for execution of a response. In other words, CNV reveals preparation for* signaled* movements and is an indicator for* anticipation*. The earlier part of the CNV is generated in response to a “Warning” cue and has maximum amplitude over the frontal cortex reflecting phase of the movement, whereas the later or terminal CNV, reflecting preparation for a motor response, begins around 1.5 s before the “Go” cue and has maximum amplitude over the motor cortex [3, 22]. The later part of CNV happens even if the subject responds at the time he anticipates the “Go” stimulus [11].

### 2.2. Generator Sources of MRCP

Several studies reported that the BP might be recorded from subcortical structures such as basal ganglia and thalamus [10]. The work [24] deduced that the early BP was produced by both sensorimotor areas. The work [25] verified that both the ipsilateral and contralateral supplementary motor areas (SMAs) generated potentials consistent with the early BP.

In order to elucidate the exact area and timing of the motor cortical activation in voluntary movement, dipole source analysis incorporating multiple constraints was applied for MRCP. The work [26] suggested that medial frontocentral (MFC) and sensorimotor areas (SM1) were probable generators of MRCP. The strength of the six dipoles, seeded at the activated spots (three dipoles in left SM1, two in right SM1, and one in MFC) revealed by fMRI, was measured over time. Inside the bilateral SM1, activation of the precentral gyrus happens bilaterally with comparable strength from −1.2 s, taken after by that of the precentral bank from −0.5 s with contralateral dominance through movement execution [26]. Consequently, the postcentral bank gets active just on the contralateral side at 0.1 s after movement. Activation of the MFC shows timing similar to bilateral precentral gyri. The strength and timing of arousal in the ipsilateral precentral gyrus were like those in the contralateral precentral gyrus and the MFC. But the ipsilateral precentral bank demonstrated much lesser strength than the contralateral precentral bank [26].

To a certain degree, automatic movements such as blinking of eyelids, spontaneous eye movements, swallowing, chewing, and respiration are also controlled by volitional factors; therefore, BP is recorded when these movements are reiterated at a self-paced rate [10]. Self-paced finger movements were related with activation of the anterior SMA, both contralateral sensorimotor cortex and the lateral premotor cortices, but without substantial activation of the ipsilateral sensorimotor cortex [27]. For externally triggered movement, a premovement potential preceding the stimulus was present [27]. Similarly, there were few distinctions in the areas of activation between externally triggered activations and self-paced activation. For a self-paced finger movement, [28] reported SMA activation anteceded that of the motor cortex by 800 ms.

The dorsal premotor cortex (PMd) is believed to play substantial role in cued movement preparation rather than in self-initiated movements [29]. The terminal CNV is generated in the prefrontal cortex including PMd, while the late BP is generated in the primary motor cortex, SMA, and primary somatosensory cortex [29]. This study discovered the effects of variation of PMd on BP and CNV reflecting self-initiated versus cued movement preparation by increasing and decreasing the excitability of brain using 5 Hz and 1 Hz repetitive transcranial magnetic stimulations (rTMS), respectively. They found that rTMS of the left PMd resulted in variation of terminal CNV but not late BP while rTMS of the SMA proper resulted in a modification of late BP but not terminal CNV. This provided evidence that neuronal activity of the left PMd in humans is favorably included in the preparation of externally cued movements as compared to self-initiated movements, contrasting with an opposite role of the SMA proper.

Comparing the MRCP for a foot movement with hand movement showed interesting differences across some movement components [30]. For the hand movement, the late BP is highest over the contralateral central area (approximately C1 or C2 of the International 10–20 System) and for the foot movement, late BP is maximal at the midline (approximately Cz) [30].

### 2.3. Recording MRCP

For the study of BP in individual subjects against hand movements, it is vital to record EEG from multiple electrodes, including C1 and C2, for identifying the abrupt increase of the gradient [30]. The main recording locations for MRCPs are C3, Cz, and C4 [31]. Different studies used different number of electrodes and locations for recording CNV, for example, C3, Cz, and C4 [32], Fz, Cz, Pz, C3, and C4 [33], and only Oz [34].

The MRCP can easily be masked by activity in the higher frequency bands because its amplitude typically lies between 5 and 30 *μ*V and only occurs at frequencies of around 0–5 Hz [31]. Several recordings of the same trials must be taken and then averaged across these trials for meaningful extraction of the MRCP from EEG traces [35]. The reason behind this approach is that EEG data recorded from a single trial contains both the MRCP waveform and spontaneous, random noise [36]. By averaging, the background noise in each trial will be cancelled out, leaving only the MRCP when the data from multiple trials is filtered to eliminate the higher frequency activity and averaged together.

### 2.4. Factors Influencing BP

Components of MRCP can be inspired by various factors such as preparatory state, level of intention, movement selection, pace of movement repetition, speed and precision of movement, praxis movement, perceived effort, force exerted, discreteness and complexity of movement, learning and skill acquisition, and pathological injuries of various brain structures. The review by [10] sums up several factors influencing BP. Recently, few studies intended to analyze the effect of kinetics of movement such as force and speed on MRCPs [37–40].

## 3. Prediction of Intention of Movement

In this section, we extracted information of the premovement or preimageries from different studies. Some studies display the usefulness of data obtained in the real-time BCIs. Studies utilized different EEG data acquisition techniques including different electrode montages and signal enhancement methods, since these are related to the results reported. Studies mentioned in Tables 1 and 2 try to answer the question whether or not the subject would like to move in the short future. These studies verify that by using MRCPs with the right EEG setting and signal processing techniques, major information can be deduced about the movement yet to come. This section briefly describes some classifiers, filters, and performance metrics used in the studies mentioned in Table 2.

### 3.1. Classification Algorithms

This section briefly describes the classification algorithms used in studies mentioned in this paper. The classification algorithms include Support Vector Machine (SVM), Linear Discriminant Analysis (LDA), Neural Networks (NN), Multilayer Perceptron (MLP), Bayesian Classifier (BC), *k* nearest neighbors (*k*NN), and Mahalanobis Distance (MD). Some of these classification techniques have been reviewed in detail by [41]. The details of Matched Filter technique can be found in [42] and for Locality Preserving Projection (LPP) in [20].

#### 3.1.1. Support Vector Machine

The Support Vector Machine (SVM) is a pattern recognition algorithm that has been successfully applied to wide variety of classification problems. It learns to distinguish among various classes of objects by some complex data transformations and then separate the data based on the defined labels for classes. For example, the data for a two-class problem consist of objects labeled corresponding to two classes, for example, +1 (data belong to class 1) or −1 (data belong to class two). The system then automatically identifies the input points and uses them to represent the solution [43].

#### 3.1.2. Linear Discriminant Analysis

The purpose of Linear Discriminant Analysis (LDA) (also called Fisher's LDA) is to use hyperplanes to isolate the data into different classes [44]. The segregating hyperplane is acquired by seeking the projection that decreases the interclass variance and increases the distance between the two classes' means. To solve a two-class problem the isolation of the input data vector into either class depends on presence of the data vector on which side of the hyperplane [44].

#### 3.1.3. Neural Networks

Neural Networks (NN) can be thought as circuits of immensely interconnected units with flexible interconnection weights, which allow us to yield nonlinear decision boundaries. They can be classified by architecture, algorithm for calibrating the weights, and the kind of units utilized as a part of the circuit [45]. Most widely used NN is the MLP.

#### 3.1.4. Multilayer Perceptron

Multilayer Perceptron (MLP) is composed of several layers of neurons: an input layer, perhaps one or many hidden layers, and an output layer [45]. Each input layer is connected with the output of the previous layer, while the neurons of the output layer deduce the class of the input feature vector. MLP can approximate any continuous function when it is composed of enough neurons and layers. It can also classify any number of classes, which makes MLP very flexible classifiers and adaptive to a variety of problems [46].

#### 3.1.5. Bayesian Classifiers

Bayesian Classifier (BC) depends on Bayes' theorem and can anticipate class membership probabilities, for example, the likelihood that a given sample fits into a specific class. In order to classify a feature vector, it learns the way of computing the probability of each class. BC assumes that estimation of a specific feature does not rely on value of any other feature, which provided the class variable. Being a generative classifier, it produces nonlinear decision boundaries and performs more efficient rejection of uncertain samples as compared to discriminative classifiers [44].

#### 3.1.6. *k* Nearest Neighbors

The objective of this method is to allocate to an unseen point the dominant class amongst its *k* nearest neighbors within the training set. *k*NN can approximate any function with enough training samples and a sufficiently high value of *k*, which allows it to yield nonlinear decision boundaries [47].

#### 3.1.7. Mahalanobis Distance

Mahalanobis Distance assigns a feature vector to a class according to its nearest neighbor(s) from a class prototype. It assumes a Gaussian distribution *N*(*μc*, *Mc*) for each prototype of the class *c*. Then a feature vector *x* is allocated to the class that links to the nearest prototype [48]:

(1) $$\begin{array}{c} d_{c}\left(\;x\;\right)=\sqrt{\left(x-\mu_{c}\right)M_{c}^{-1}{\left(x-\mu_{c}\right)}^{T}}. \end{array}$$

### 3.2. Spatial Filters

A spatial filter amalgamates data from two or more locations (electrodes). Spatial filtering techniques comprise common spatial patterns (CSP), common average referencing (CAR), surface Laplacian (SL), independent component analysis (ICA), and principle component analysis (PCA). This section briefly describes some spatial filters; for more details please refer to [49].

ICA is a method intended to find a linear illustration of non-Gaussian data in the form of statistically independent constituent components [50]. Measured signals comprising a linear mixture of statistically independent source signals can be dissolved into their vital Independent Components (ICs), hence deducing the original source signals using ICA [50, 51]. ICA finds the weighting of the channels from the data like PCA and CSP [4], while CAR and SL amalgamate channels linearly to produce a set of weights that does not depend on the underlying data [4]. SL highlights the radial component of the neural activity placed directly below each recording electrode from sources, whereas ICA is capable of detecting both radial and tangential sources and consequently may be beneficial over SL [4, 52]. The details regarding CSP and CAR can be found in [49].

### 3.3. Performance Measures

The performance of studies is computed using sensitivity, specificity, and detection error. Sensitivity (also known as true positive rate (TPR)) quantifies the fraction of actual positives (movements) which are precisely recognized. Specificity (also called the true negative rate (TNR)) assesses the fraction of negatives (no motion or noise) which are exactly detected. Sensitivity and specificity are calculated using the following equations, respectively, where TP and TN represent number of true positives and number of true negatives, respectively [53]:

(2) Sensitivity=TPTP+FN,

(3) Specificity=TNTN+FP.

## 4. Studies for Predicting the Intention of Movement

For the development of self-paced closed loop BCIs, the robust detection of motor intention is a vital and critical issue. In the past decades, sensory motor rhythms have been used for detection of motor intention in studies comprising BCIs to control visual feedback [54] or trigger external devices [55] without investigating latency. In the initial studies, the acceptable delay in control has not been considered in detail for BCI control applications. In other fields, for example, multifunction prostheses control by myoelectric signals, a 200 ms delay is considered acceptable [56–58]. To induce plasticity in BCI-based neurorehabilitation applications, it was demonstrated that the required delay was in the same range as for control, that is, in the order of a few hundred milliseconds [59]. Therefore, a reliable detection with minimal latency and high accuracy would play a vital role in an effective BCI rehabilitation tool [7].

### 4.1. Techniques Utilized and Performance

In recent years, slow cortical potentials captured the attention in the rehabilitation field. Several studies have been reported, which concentrated their attention intended for communication purposes.

Yom-Tov and Inbar [43] developed an algorithm combining Matched Filter, a nonlinear transformation, and a classifier to detect MRCP using small number of EEG channels. The algorithm was compared with Mason-Birch low-frequency asynchronous detector (LFASD) and optimum detector by both offline evaluation and theoretical analysis. The algorithm used by this study showed 25% improvement and the detector worked at a rate of 25 decisions s^−1^ as compared to 16 decisions s^−1^ in the LFASD. But the detector may not be useful in every application and failed to operate correctly, partly due to interference of MRPs from other limbs of the body and possibly due to imagined movements. These verdicts indicate that correctly identified features have a major role not only in discarding MRCPs due to movements that are not part of the BCI system, but also in determining the limb moved (or imagined movement) by the user. During sessions, training the subject to reduce body movement can probably attain better results.

To detect movement planning, [60] employed a user-specific template matching structure as part of a method. In this study, the emphasis was more on movement detection than the prediction. Performing actual finger movement with cues, one electrode recorded BP waveform which was then used to build the template. However, inconsistency in performance between subjects was evident in this study which needs more investigation or perhaps a different methodology.

From single trial EEG, [61] showed that effectual combinations of computational methods can deliver possible classification of human movement intention using large number of electrodes. The combinations of temporal filtering using power spectral density estimation and discrete wavelet transform, spatial filtering using ICA and surface Laplacian derivation, and classification methods using LMD, QMD, BC, and SVM provided higher performance than those of other combinations. Evaluation is recommended in order to check whether performance can be enhanced after training with feedback or not.

The validity of OSF on imagination of isometric plantar-flexion was confirmed in a study conducted by [53]. Features were extracted with PCA and classification was performed using *k*NN and SVM. In this study, the TPRs were high (80–90%) but the method was verified on segmented data instead of ongoing EEG traces with one subject only.

Kato et al. [34] designed a BCI master switch by detecting the CNV related potentials and performed both offline and online studies. In order to ameliorate the single-trial discrimination of user intentions to switch, CNV was employed due to its high SNR. Using only one electrode and performing four cued button press tasks, they also applied SVM to improve the single-trial detection of CNV-related potentials. Their online system did not discern between “intend to switch” and “do not intend to switch.” This was maybe because of using default parameters of SVM in LIBSVM for the distinction of CNV-related potentials [34, 62].

The detection of movement intention from single trial MRCPs of movement imagination and movement execution was performed by [42]. The task performed by the subject was always the same (ankle dorsiflexion). They performed offline detection due to instrumentation limitation and provided the feasibility of the approach in stroke patients, along with the extensive analysis in healthy subjects. The accuracy of the detection of movement intention was measured by applying a similar spatial filtering technique. In this study, a portion of the negative phase (2 s) of the MRCP was used as a template. In order to improve SNR of MRCP, OSF was used (TPR of 82.5 ± 7.81%). OSF outperformed large Laplacian spatial filter (TPR of 68.7 ± 14.9%) and CSP (TPR = 55.4 ± 14.01%).

Lew et al. [63] explained that it is possible to predict the movement 500 ms before its occurrence. For the training phase, the signal prior to movement onset by 500 ms was used in comparison with 500 ms before the auditory cue. While a shifting window was implemented for the test phase and LDA was employed, their results showed maximum average TPR of 81% for left hand while 79% for right of stroke and control subjects. While for healthy subjects average TPR was 76 ± 7% with latency of −167 ± 68 ms. This offline study employed large number of electrodes with small sample size of patients.

Ahmadian et al. [64] showed the superiority of CBSE based algorithm in detection of brain potential compared with BSS based algorithm using LDA. Subjects performed cued button preprocessing. CBSE based algorithm took 0.26 s while BSS based algorithm took 51.90 s. All 128 channels EEG data was employed in the analysis. It was suggested that false detection rate can be reduced if BSS-based algorithm uses extracted sources which are mistaken with the shape of BP from other regions of brain. On the other hand, this amendment would increase the computation time.

Motor intention could be detected from MRCP using the Matched Filter, with small latency and satisfactory accuracy. The same task was performed in [19, 20, 42, 65]. Niazi et al. performed analysis on both healthy and stroke patients [42, 65]. He investigated the possibility of eluding the individual training phase in the detection of movement intention. The detection accuracy with the average template for the motor imagery data was 65 ± 22% [65] and with the individual template was 60 ± 13% [65].

Jochumsen et al. [39] detected movement intentions and extracted distinct levels of speed and force of the intended movements. The temporal features were classified with an optimized SVM. This study evaluated performance when detection was combined with classification. The system correctly detected 81% of the movements. At the point of detection, system classified 75 ± 9% and 80 ± 10% when altering the force and speed, respectively. After combination of detector and classifier, the system detected and correctly classified 64 ± 13% and 67 ± 13% of movements. Incorrectly detected and classified movements were about 21 ± 7% and 16 ± 9% while latency was 317 ± 73 ms before the movement onset. The authors included only healthy subjects and the signals were processed offline. The performance of system will decline if user is a patient due to severity of motor impairment, mood, and amount of training.

Xu et al. [20] performed analysis on healthy subjects only. In this study, LPP-LDA showed higher accuracy and shorter latency than Matched Filter, having limitation that the classifier would not work when training trials were less than 15. The proposed algorithm had similar FPR for imagination and execution across all subjects. TPR for execution and imagery was greater than 80% and 70% in this study, making it significantly better than those with Matched Filter approach [19, 42, 65]. Also detection latency (315 ± 165 ms) was significantly shorter than that with Matched Filter (460 ± 123 ms) [19, 42, 65].

To further improve the results, [66] applied ICA and the LSF to improve the signal-to-noise ratio of MRCP. Following these preprocessing steps, Matched Filter was applied to perform single-trial detection of gait initiation. TPR was 76.9 ± 8.97%, and the false positive rate FPR was 2.93 ± 1.09 per minute. On a single trial basis, these results demonstrated the possibility of detecting the intention of gait initiation from EEG signals.

In a recent study by [67], six healthy persons and eight stroke patients performed upper limb self-paced reaching movements. This study used a classifier that combined the information acquired from analysis of the BP and ERD cortical processes. System validation was performed with the combined classifier (ERD and BP patterns) and equivalent classifiers (using either BP or ERD). The results obtained for healthy subjects were similar to [20]. However, the average latencies (healthy: −89.9 ± 349.2 ms and patients: 35.9 ± 352.3 ms) were less than [20] (315 ± 165 ms). These dissimilarities might be due to differences in the way subjects executed the task in each experiment such as changes between upper limb and lower limb cortical patterns, speed of movements among others, and length of the resting intervals between movements. The observed alterations could also be because of the combined use of the ERD and BP features anticipated here, which facilitates reduction in FPR and, as a result, enables the preference of more anticipative detection thresholds [67].

A complexity confronted in this paper involves the absence of similar studies in terms of purpose of detector, signal acquisition, limb movement, and number of electrodes. It should be noted that movements executed in different studies were not similar leading to variances in signal morphology and SNR. Ideally studies should be compared within the same context, that is, with similar protocol, users, and similar extraction of features.

Although the mentioned studies have delivered a valuable insight into the prediction of MRCPs using different signal acquisition techniques, the framework of research is not without its impediments. One limitation relevant to most of the studies mentioned is the absence of clear ecological validity in the research, that is, “the extent to which an experimental situation mimics a real world situation” [31]. Presence of an ecological validity in a study means that the expertise used in the laboratory situation could be as equivalent as possible to the real skill the research is exploring and preferably identical.

### 4.2. Guidelines to Choose a Classifier

EEG signals are notably nonstationary so training sets acquired from different sessions are probable to be quite different. Consequently, a low variance (sensitivity to training set) can be a solution to tackle with the variability issue in some studies. Unstable classifiers tend to have a low bias (deviation between the estimated mapping and the superlative mapping) and a high variance, while stable classifiers have a high bias and a low variance [41]. This might be an explanation of why some simple classifiers like SVM, Matched Filter, and so forth sometimes surpass more than complex ones. Simple classifiers are generally slower than other classifiers but fast enough for real-time applications. Here the question is whether it is worthy to get higher performance at the expense of computational cost. In order to attain minimal classification error, both the variance and the bias must be small. Unfortunately, natural variance-bias tradeoff is always present [41].

The classifier will probably give bad performance if the number of training data is lesser matched to the size of the feature vectors. Usage of at least five to ten times training samples per class as the dimensionality is recommended [68, 69]. Generally the training set is small and dimensionality is high so; unluckily this cannot be useful in all BCI systems. One of the reasons might be the long duration of the tasks, which on the other hand is hectic for subjects. Consequently this “curse” is a key concern in BCI design.

Furthermore, combinations of classifiers also seem to be very efficient in some studies [20, 70, 71]. Normally experiments are performed in a controlled manner minimizing noise and other artifacts while presence of noise in real life scenario is quite obvious. One possible solution might be to increase the generalization abilities of the classifier.

## 5. Future Work

MRCP has been employed as a control signal in BCI technology. It is mainly beneficial for neuromodulation applications in which the delay between the intention of action and the feedback from the system is crucial to induce plasticity [19]. BCIs have primarily been used for control and communication purposes [4]; however, in recent years its prospective in neurorehabilitation has been studied such as functional electrical stimulation [6]. BCIs are extensively used in research and major concern is its long-term effects or long-term changeability of EEG signals to evaluate retention of the plasticity over time [7]. So there is a need to design studies over longer duration to evaluate the performance and accuracy of the BCI system for healthy subjects and patients. As signal processing in BCIs continues to progress, the next perspective is to integrate additional information regarding neurophysiology, disease behaviors and its advancement, and signal dynamics into the existing or future approaches.

## 6. Conclusion

EEG data collected prior to imminent movement which associates with motor preparation and planning period of the brain present substantial prediction potentials. Illustrating the intention to move through MRCP can be employed in rehabilitation protocols. Depending on the purpose of the BCI system, a higher TPR could be achieved increasing the number of false positives, while some studies tend to give a priority to a low FPR rather than high TPRs [43]. In summary, this paper reviews the proficiency of EEG in predicting the next motor task and primarily targeted at providing the examples of the progress in this field.

## Figures and Tables

**Table 1:** Experiment protocols of studies reviewed.

Reference	Number of subjects	Number of electrodes	Movement type	Self-paced or cue-based	Brain signals
(Yom-Tov and Inbar, 2003) [43]	5 (healthy)	9, 4 out of 9 channels were used	Executed finger movement (button press)	Self-paced	MRPs

(Haw et al., 2006) [60]	5 (not mentioned)	1	Executed finger movements	Cue-based	BP

(Bai et al., 2007) [61]	12 (healthy)	122	Executed hand movement	Self-paced	MRCPs and ERD (event-related desynchronization)

(Boye et al., 2008) [53]	1 (not mentioned)	9	Executed and imagined foot movement (isometric plantar-flexion), but only imaginary task was further analyzed	Cue-based	MRCP

(Kato et al., 2011) [34]	7 (not mentioned)	1	Executed and imagined finger movements (button press)	Cue-based	CNV

(Niazi et al., 2011) [42]	19 (healthy) and 5 (stroke patients)	10	Executed and imagined foot movement (ankle dorsiflexion)	Self-paced	BP

(Lew et al., 2012) [63]	8 (healthy), 2 (control), and 2 (stroke patients)	64, 34 out of 64 channels were used	Executed arm movements (reaching task)	Self-paced	BP

(Niazi et al., 2012) [19]	16 (healthy)	10	Imagined foot movements (dorsiflexion)	Self-paced	MRCP

(Niazi et al., 2013) [65]	20 (healthy) and 5 (stroke patients)	10	Executed and imagined foot movements (dorsiflexion)	Self-paced	MRCP

(Ahmadian et al., 2013) [64]	3 (healthy)	128 channels	Finger movement (button press)	Self-paced	BP

(Jochumsen et al., 2013) [39]	12 (healthy)	10	Executed foot movement (isometric dorsiflexion)	Cue-based	MRCP

(Jiang et al., 2015) [66]	9 (healthy)	9	Executed foot movements (stepping)	Self-paced	MRCP

(Xu et al., 2014) [20]	9 (healthy)	9	Executed and imagery foot movements (dorsiflexion)	Self-paced	MRCP

**Table 2:** Techniques used for prediction of onset of movement and main findings of the studies reviewed.

Reference	Preprocessing techniques	Classifiers	Performance	Latency (ms)	Offline or online system	Single-trial analysis	Limitations
(Yom-Tov and Inbar, 2003) [43]	Low-pass filter (10 Hz) using 8th-order Chebyshev	Simple threshold element, support vector machine (SVM), and linear vector quantiser 3-feature reduction with 1-nearest neighbor (1-NN)	Using hybrid detector 25% improvement in performance was achieved as compared to Mason-Birch low frequency asynchronous detector (LFASD)	25 decisions s^−1^	Offline	—	Detector fails to work correctly partly due to MRPs related to other limbs and imagined movements

(Haw et al., 2006) [60]	Building a specific template during 3 or 4 training sessions for each subject	Thresholding based on correlation and error	Accuracy was 70% with a false positive rate (FPR) of (5/24)	—	—	Yes	Variability in performance between users

(Bai et al., 2007) [61]	Low pass filter (100 Hz) using 3rd-order Butterworth filter	Linear Mahalanobis Distance (MD), Quadratic MD, Bayesian Classifier (BC), Multilayer Perceptron (MLP) Neural Network, Probabilistic Neural Networks, and SVM	Accuracy was 75%	—	Offline	Yes	Large number of electrodes (122)

(Boye et al., 2008) [53]	Downsampling from 500 Hz to 20 Hz, with antialiasing prefiltering (0–5 Hz) and PCA and Locality Preserving Projection (LPP)	A variation of *k*NN and SVM	Sensitivity for SVM = 96.3 ± 2.0% for *k*NN = 84.5 ± 5.1%; specificity for SVM = 94.8 ± 2.7% and for *k*NN = 98.9 ± 1.2%	—	—	Yes	Method was tested on segmented data rather than ongoing EEG traces with only 1 subject

(Kato et al., 2011) [34]	Low pass filter (35 Hz) and high pass filter (0.05 Hz) for EEG and 0.1 Hz for EOG	SVM	Detection rate (intention to switch = 99.3% and (not to switch = 2.1%)	—	Both	Yes	Online system cannot differentiate between intend to switch and do not intend to switch

(Niazi et al., 2011) [42]	Band pass filter (0.05–10 Hz) with Optimized Spatial Filter (OSF)	Neyman Pearson Lemma	For healthy subject's movement execution TPR = 82.5 ± 7.81% and for movement imagination TPR = 64.5 ± 5.33%	−66.6 ± 121	Offline	Yes	Small sample size (patients) and no online detection due to instrumentational limitation
			For stroke patients TPR = 55.01 ± 12.01%	−56.8 ± 139			

(Lew et al., 2012) [63]	Narrow band zero phase noncausal IIR filter with cutoff frequencies of 0.1 and 1 Hz	Linear Discriminant Analysis (LDA)	TPR = 76 ± 7% (healthy)	−167 ± 68 (healthy)	Offline	Yes	Large number of electrodes (34)
			For stroke and control subjects TPR = 81 ± 11% (left hand) versus (right hand) TPR = 79 ± 12%	Right hand = −140 ± 92 versus left hand = −162 ± 105			

(Niazi et al., 2012) [19]	Band pass filter (0.1–100 Hz) and OSF	Matched Filter	TPR = 67.15 ± 7.87% and FPR = 22.05 ± 9.07%	−125 ± 309 (offline)	Online	—	Different aspects of triggered stimulations were not fully considered

(Niazi et al., 2013) [65]	Band pass filter (0.05–10 Hz) and OSF to maximize SNR	Matched Filter	For motor execution (healthy) TPR = 69 ± 21% and FPR = 2.8 ± 1.7	−196 ± 162	Offline	Yes	—
			For stroke patients TPR = 58 ± 11% and FPR = 4.1 ± 3.9	152 ± 239			
			For motor imagery (healthy) TPR = 65 ± 22% and FPR = 4.0 ± 1.7	—			

(Ahmadian et al., 2013) [64]	Filtering data between 0.1 Hz and 70 Hz	Independent component analysis (ICA)	Computation time for constraint blind source extraction (CBSE) algorithm was 0.26 s and blind source separation (BSS) algorithm took 51.90 s	260	—	Yes	Large number of electrodes (128) with small number of subjects

(Jochumsen et al., 2013) [39]	Band-pass filter (0.05–10 Hz) using 2nd-order Butterworth in forward and reverse direction with three spatial filters, large Laplacian spatial filter (LLSF), OSF, and common spatial patterns (CSP)	SVM	TPR = ~80% and FPR <1.5 accuracy = 80 ± 10% (speed) and 75 ± 9% (force)	317 ± 73	Offline	Yes	Inclusion of only healthy subjects

(Jiang et al., 2015) [66]	ICA followed by LSF to enhance SNR	ICA	TPR = 76.9 ± 8.97% and FPR = 2.93 ± 1.09 per minute	−180 ± 354	Offline	Yes	Prediction of gait initiation was not done

(Xu et al., 2014) [20]	Band-pass filter (0.05–3 Hz) and large LSF to enhance SNR	LPP followed by LDA	LPP-LDA TPR = 79 ± 12% FPR = 1.4 ± 0.8 per minute	315 ± 165	Online	—	Inclusion of only healthy subjects and classifier did not work for training trials less than 15
